# Text-Book of Opthalmalogy

**Published:** 1893-07

**Authors:** 


Text Book of Opthalmalogy, by Dr. Ernest Fuchs. Translated by Dr. A. Duane, of New York. D. Appleton & Co.

This is undoubtedly, everything considered, the most complete text-book on Opthalmalogy up to date. We are as much under obligations to Dr. Duane, the translator, as to the author, for his accurate and approved adaptation of the original text.

Its typographical features are without a blemish, which, however, would go without the saying, as it comes from the Appletons, since we have so long expected nothing else from their hands.

